# Mine Land Reclamation and Eco-Reconstruction in Shanxi Province I: Mine Land Reclamation Model

**DOI:** 10.1155/2014/483862

**Published:** 2014-06-22

**Authors:** Hao Bing-yuan, Kang Li-xun

**Affiliations:** Taiyuan University of Technology, Taiyuan, Shanxi 030024, China

## Abstract

Coal resource is the main primary energy in our country, while Shanxi Province is the most important province in resource. Therefore Shanxi is an energy base for our country and has a great significance in energy strategy. However because of the heavy development of the coal resource, the ecological environment is worsening and the farmland is reducing continuously in Shanxi Province. How to resolve the contradiction between coal resource exploitation and environmental protection has become the imperative. Thus the concept of “green mining industry” is arousing more and more attention. In this assay, we will talk about the basic mode of land reclamation in mine area, the engineering study of mine land reclamation, the comprehensive model study of mine land reclamation, and the design and model of ecological agricultural reclamation in mining subsidence.

## 1. Introduction

As is well known, coal is the foundation of national economic development and is also one of the main primary energies in China. In our country the coal production shows rapid growth rate. For example, the output was 2960 million tons in 2001, while the yield grew up to 1381 million tons in 2009 with an average annual growth rate at 175 million tons [1]. The rapid development of the coal production meets the requirements of social and economic development and is the important foundation of national economic development [2]. It accounts for about 75.6% and 68.7%, respectively, in energy production and consumption structure in 2008.

The main position of coal remained unchanged for nearly 50 years. The coal plays special important roles in the process of industrialization, urbanization, and modernization in our country. However, the mining, transportation, and utilization of coal bring up a series of environment problems, as is the trouble we have to confront and solve in building a well-off society and achieving good ecological mining area [3].

All the mining activities will lead to subside, exert an effluence on surface structures and environment such as reduction in farmland output, flatlands ponding, road crack, and house collapse [4]. According to statistics, the forest broken directly by mining added up to 1060 thousand hectares, the destroyed grassland 263 thousand hectares [5]. In addition, the area of collapse due to the mining industry reached 5000–6000 thousand mu including farm land 1300 thousand mu, about 40 cities. The cities all suffered from mining subsidence and 25 cities of them were seriously damaged. Every year, the loss on account of mining subsidence reached more than 400 million. In short, the damage brought about by mining subsidence is more serious than earthquake [6]. Shanxi Province is an important coal resource province in our country. It was reported that its coal reserves accounts for 1/3 in the total reserve in our country, the coal production for 1/4 in national total output, coal transfer volume per year for 3/4 in national inter provincial volume and coal export volume for 1/2 in the national total exports [7]. In face of the narrow agricultural base, worse circumstance, and lack of arable land and resource per person in Shanxi province, how to resolve the contradiction among coal resource exploitation, subsidence, and environmental protection have become the urgent affairs [8].

Recently, the idea of green mining and mining industry were proposed in succession and got widely recognized. One of the core concepts of the “friendly mining industries” was to achieve the “green mining” [9]. The connotation of the green mining was to strive to follow the principle of the green industry in economy, forming a “low mining-high use-low emissions” production technology [10]. Namely, we should make full use of the available resource exploitation and ecological environmental protection technology as well as geological conditions to realize the mining development model with optimal economic benefits and minimal ecological environmental impact [11]. In this model the main point focuses on the scientific and reasonable control of the subsidence in coal mining. The unreasonable exploitation of the coal district will lead to a series of environmental problems in the following.

1. The mining subsidence in coal field severely damaged the mining area environment, resulting in the house crack, farmland ponding, road fracture, pond dried up, and so on. All the damages make a great difference and loss to daily life and production. The destruction of floorscape is specially difficult to recover.
2. The subsidence caused damages to buildings, roads, railways, bridges, underground pipe network, and urban infrastructure, leading to railroad and road diversion, reconstruction of the buildings, and huge economic losses.
3. The subsidence destructed the groundwater level and water system, forming low-lying area and pools zone and even wetland. The destroyed land neither can be used for planting nor breeding turning into wasteland in the end. The destruction of the underground water system and the run off of the underground water are unrecoverable.
4. The subsidence disrupted the natural forest, grasslands, vegetation, and massif, affecting the ecological balance [12].
5. The subsidence can also influence the industry-agriculture relation and social stability, sharpening the contradiction between enterprise and local regions and aggravating social instability.

Based on the above problems, this paper conducted the study about the utilization model of mine land reclamation.

## 2. The Basic Mode of Land Reclamation in Mine Area

The use of mine land reclamation mainly includes agriculture, forestry, fishery, construction, and entertainment [13].

### 2.1. The Reclamation for Agriculture

According to the Land Administrative Laws, the reclaimed land should on a priority basis be used for agriculture. The agricultural reclamation can adopt different management methods in accordance with different situations. On account of the rugged areas in mountainous regions, the shallow subside can be used for reclamation directly with a little proper adjustment [14]. In plain areas, the shallow subside which was formed can be transformed into dry land or paddy field, respectively, according to the water table [15]. As for the deep collapse with a low water table, we should take the fill and overlying transformation methods. Finally, with regard to the extensive, deep, and low water table collapse, we have to do comprehensive treatment. Generally, we took the method of digging, filling, and filling the rimland before reclamation.

### 2.2. The Reclamation for Forestry

The reclamation of sank land for forestry is decided by the terrain and the soil property [16]. In general, the asperous land in hill and mountainous regions, and the irregular reclaimed land are suitable for garden. In these gardens we can cultivate cash crops or do afforestation. The land which is barren, elevated, and steep should be reclaimed into forest or meadowland [17]. For the collapse in low mountains and hills or overlying land after strip mining, they are fit for planting forages or trees [18].

### 2.3. The Reclamation for Fishery

It is cheaper and faster to perform fishing reclamation. Normally, the land sank in plain with ponding at the 2 meter or more deep can be transformed into a fish farm. The water accumulated area for all year round can carry out fish farming in the depth of the subsidence [19]. The subsidence areas which are excavating coal are still collapsing, so we can adopt the extensive reclamation mode to do fish-duck polyculture.

### 2.4. The Reclamation for Construction

The leveled mining area in the subsidence region can be used for building. The areas after foundation treatment can be used to construct residential houses and industry building. This measure on the one hand can relax our country land to be extensive to some degree, and on the other hand can offer nearby place for the countryside transfer, getting around the move again, relieving the relationship between industry and agriculture, saving compensation for damages and the cost of relocating again [20].

### 2.5. The Reclamation for Tourist

The filled collapse should be smartly planned. We can set tourist attractions, plant trees and flowering straws with ornamental value, and build ponticulus and lakes for fish and sightseeing [21]. The collapse with large water surface, deep water, and good water quality can build underwater park or amusement center.

### 2.6. The Reclamation for Headwaters

The subsidence basin should be able to store and block up water as much as possible under the rational development and utilization. The deposit water can supply underground water or conduct aquaculture or irrigate land. For the north which lacks water, this mode is a very good development approach for water source. The collapse areas with good quality water can be developed into a newly waterhead area. We can also build water works in these regions. The purification treated hydrops is transformed into drinking water to relieve the lack of water in urban, residential areas and company.

Technical requirements of the land reclamation mode are shown in Table 1.

## 3. Engineering Study of Mine Land Reclamation

Since the mid-1980s, the reclamation technologies including gangue filling, flyash filling, and digging deep to fill shallow achieved success in Huaibei coal mine area. The Xuzhou coal mine also acquired the successful experiences in land formation, drainage, reducing immersing, and ecological engineering reclamation [22]. The other coal mines obtained technology experiences such as salt marsh governance, waste heap reclamation for afforestation, collapse pit for open mine, and building reclamation [23]. For example, the comprehensive treatment technology method study of subsidence reclamation in Huaibei coal mine, the mine land reclamation study in Tongshan county, and the study of waste heap reclamation in Hegang mining area.

The acquirement of all the above experience and achievement can be said to set the style for mine land reclamation in our country, making a pioneered contribution.

### 3.1. Land Formation and Gradient Technology

This technology is suitable for low water table mining areas or the middle-high water mining areas which are implemented with drainage and reducing immersing measures [24]. The following should be noticed in applying this method: the stripping and preserve of the topsoil, the determination of the elevation after land leveling, the determination of elements in the terrace section, the mating of the irrigation-drainage methods, and so forth.

**Figure 1:**
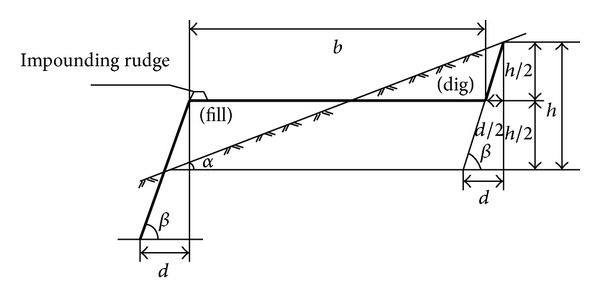
Slope-to-terrace sectional view.

As for the collapse areas without hydrops, the side of the ponding collapse and the collapse areas in hills, the reclamation methods of land formation and transformation into terrace or greenbelt are suitable for all. Slope-to-terrace sectional view is shown in Figure 1. Commonly, the additional gradient produced by mining subsidence is minor. The collapsed ground with the slope gradient within 2° can be used for farming after land leveling. The collapsed ground with the slope gradient between 2°–6° can be transformed into terrace according to the topographic contour and it should be cambered inwards to dam up water for soil moisture. Adopting contour ploughing mode in farming is in favor of soil and water conservation.

### 3.2. Elevating Land-Reclamation Technology

The main stuffing in elevating reclamation includes gangue, fly ash, hydraulic clay, household refuse, industrial refuse, and out-soil backfill. The better choice of elevating reclamation is to use mud in pond, lake and river, industrial refuse, out-soil, and so on. The measure should be taken to prevent secondary pollution if the gangue, fly ash, and industrial refuse were used [25].

#### 3.2.1. Gangue Filling Reclamation Technology

The approach of gangue filling reclamation technology is appropriate for all kinds of mining areas [26]. The gangue can not only act as the filling material to renew the destructive land, but also can reduce the covering and pollution of gangue. The reclaimed land can be used for agricultural as well as building land.

The steps of gangue reclamation are as follows: in the stable subsidence areas, the superficial mellow soil should be peeled and stacked around at first, and then the gangue is filled. Once the filled gangue reaches horizontal, the mellow soil should be covered on the gangue, acting as plowing layer. Isolation treatment is required in the reclamation if the gangue is poisonous and harmful. Namely, lay the water-proof layer of clay in the collapse pit to prevent the drain of the poisonous and harmful material in gangue. The gangue can be set directly in the collapse for reclamation when the collapse is not taking shape or stable. That is, predict the areas to submerge and take out the topsoil to pile up around, and then set the gangue by the dumping equipment in advance according to the predicted subsidence depth and scope. Finally, cover the topsoil which is stacked all around on the gangue layer to backfill when the gangue is filled to the anticipated level.

For the gangue reclaimed land which is used for farming, the filled gangue should be dense at the bottom and loose at the upper part so as to conserve soil moisture and nutrient to facilitate plant growth. For the gangue reclaimed land which is used for building, the handling should be performed in accordance with demand.

The elevation of the land reclamation in collapse should be determined by the application, groundwater elevation, and flood elevation. Generally, the reclaimed elevation of the building land should be higher than the local flood elevation or the restored original elevation. With an eye on the decline of the surface and the water table after exploit, the reclaimed elevation of agriculture and forestry is always lower than the original elevation in order to facilitate the soil moisture, water and fertilizer preserving, as well as the growth of crops. Therefore, the reclaimed elevation of agriculture and forestry is decided by the water table in the reclamation area. If the reclaimed surface exceeds the elevation of the water table, it should be confirmed by the water logging tolerance of the crops.

**Figure 2:**
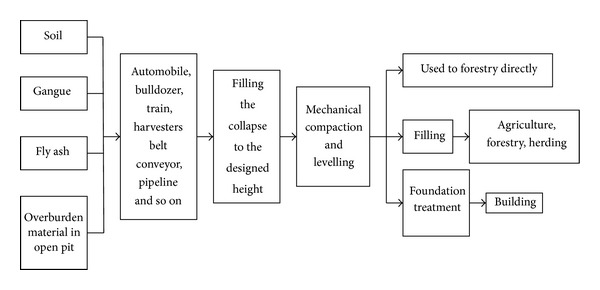
The industrial and mining area filling reclamation process flow diagram.

The technological process of industrial and mining area filling reclamation is shown in Figure 2.

#### 3.2.2. Drainage Reclamation Technology

In the diggings which are plain or have higher water table, the land surface subsidence is always accompanied by ponding or undischarged water, hindering cultivation. The surface ponding can be divided into two cases as follows.

1. The flood level of the outside river is higher than the surface elevation, and thus the water in the collapse cannot drain out automatically. Filling reclamation or exhausting the ponding in the collapse can be applied to remould the areas for farming.
2. The flood level of the outside river is lower than the surface elevation, and thus the water in the collapse can flow automatically. Now a suitable drain system is needed to automatically discharge the ponding in the collapse. In addition, if the groundwater table is overtop, draining channel is needed to lower the underwater level to guarantee the normal growth of the crops.

The key of the drainage reclamation is the design of the drainage system. Due to the integrality of the water, the situation of the whole mine or even the whole digging should be taken into consideration to form a comprehensive drainage system in designing the drainage system. Therefore, we should design the drainage system with a global idea.

Normally, the drainage system is made up of drainage ditch and impounding facility, drainage receiver outside the drainage area, and the drainage hub. The fixed channel of drainage ditch is divided into four levels according to the scope and effect of the discharge. The impounding facilities can be lake, swag, reservoir, and so on, and the drainage ditch can also be used for water impounding. The drainage receiver is the so-called outside river. The drainage hub is referred to the drain valve, powerful drainage station, and so on.

#### 3.2.3. The Utilization of Water Surface and Eco-Engineering Technology

The mining areas with high ground-water level have higher probability of hydrops, and thus the commercial and effective reclamation technology for these regions are to combine the utilization of water surface and ecological engineering. The significance of this technology manifested on one hand in that we need not to invest a huge sum of money on eliminate stagnant water and on the other hand in that the reserved water surface has great ecological value in maintaining the ecological balance of mining areas. For example, the collapse pit can be used for flood storage for irrigation. The water park has ornamental value and the scientific utilization of the water surface is able to acquire high economic benefit and so on [27].

It should be noticed in applying this technology that we should take advantage of the ecological engineering technology as much as possible to increase the production of the aquatic animals and plants, and thereby the production capacity of the waters and the productivity of the reclaimed land are improved.

### 3.3. The Dynamic Prereclamation Technology in Industrial and Mining Areas

This technology is mainly used to settle the problems of the reclamation in unstable collapse areas [28]. It is performed when the collapse is not complete and the hydrops has not formed. This technology designed according to the scale, scope, and goal of the reclamation is composed of two stages: the calculation of the engineering design and the implement of the engineering. The calculation of the engineering design includes the calculation of the expected deflection, the partition of the construction field, and the determination of the construction parameter. Compared to the commonly used technology “destruction first, government later,” this technology has the following advantages: the dynamic prereclamation achieves the effective combination of mining and reclamation, reducing the reclamation input, shortening the reclamation cycle, augmenting the reclamation benefit, promoting the sustainable utilization of the land resource in mining areas, and achieving the sustainable development of the mine areas. All the advantages can effectively relieve vegetation deterioration in mining, ecological deterioration resulted from water loss, and soil erosion, protecting the land resources at the maximum level.

**Figure 3:**
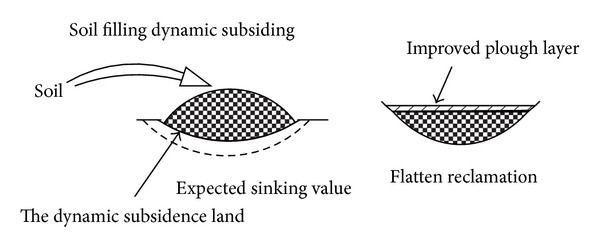
The dynamic prereclamation schematic diagram of soil backfilled collapse.

**Figure 4:**
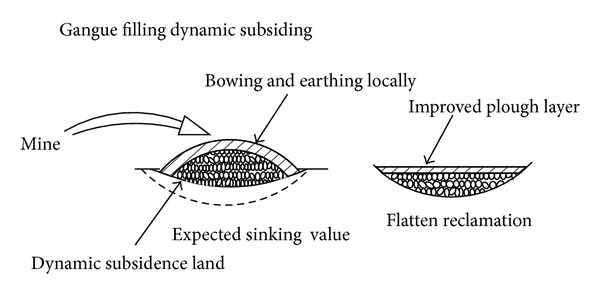
The dynamic prereclamation schematic diagram of gangue backfilled collapse.

The dynamic prereclamation drawing is as shown in Figures 3 and 4.

### 3.4. The Technology for Combing Reclamation Measures with Mineral Engineering

The combing of reclamation measure and mineral engineering may take the following forms: the field with bad coal seam occurrence conditions and coal quality should take the local mining to reduce the damage to fertile farmland. Construct a reasonable system of discharging gangue to transport gangue directly to the collapse. Underground mining should make a good mining project (such as the mining sequence and mining area partition) to reduce the loss on the floor. The opencast working should optimize the mining technology by casting mining to reduce the dumping coving and to do the reclamation for farming. The mining waste land should be used for coal mine capital construction as much as possible.

### 3.5. The Land Improvement and Utilization Technology following Reclamation

The primary mission of the biological reclamation is to improve the reclaimed soil and optimize the direction of land use. The method of improving the reclaimed soil should consider the forms and physicochemical property of the reclaimed soil, material, and technological conditions in diggings. Moreover, the methods should combine with planting strategies, too. The land-using technology not only includes the primary determination of the land utilization but also contains the planting plan and cultivation methods in recovery period of soil fertility.

The improvement measures for different reclaimed soil are displayed in Table 2.

## 4. The Comprehensive Model Study of Mine Land Reclamation

### 4.1. The Model of Gangue Filling Reclamation for Farmland in Mining Subsidence

In this model, the stowed discharge of gangue is transformed into pile up in stable collapse for filling reclamation, or to pile up in unstable collapse for prefilling reclamation. Finally, the agriculture is performed on it.

In this model the filled reclamation should be partitioned and reasonable, realizing divisional filling and divisional backfill cycle.

### 4.2. The Model of Fly Ash-Filling Reclamation and Eco-Reclamation in Subsidence Area

Now, the fly ash reclaimed land is mainly used for agroforestry planting. The covered earth should be over 0.3 meter at the thickness and the planted crops grow well [29]. Generally, the bore fruit on the land meets the demand of the sanitary standard. For the reclaimed land with higher fluorine, it should be used for cultivating trees which do not take part in the food cycle. With the comprehensive development of relocated pressing coal villages, more and more fly ash reclaimed land is used for resetting village foundation. In order to endow the fly ash reset village with long-term stability, we should study the fly ash processing technology actively [30]. We can predict that relocating the pressing coal village on the fly ash reclaimed land is going to be an important approach to settle the coal mining in village with high underground water level.

### 4.3. The Comprehensive Model of Agroforestry Reclamation in Nonponding Collapse

The nonponding collapse on plain has deep layers, fertile soil, and abundant ground water resources. Although the surface is rugged, the soil layer does not change significantly and the change in soil nutrition is small. The reclaimed land can restore the intrinsic value-in-use as long as we take engineering measures to level land and improve the water conservancy conditions. The specific engineering measures are (1) land leveling project, and (2) restore road, conservancy conditions, and agroforestry net.

The nonponding collapse in mountains and hills is usually reclaimed by land leveling, transforming into terrace or terrace green belt along the topographic contour. The key of the technology is the determination of the terrace section element, the determination of the land leveling elevation, the mating of the drainage and irrigation facilities, as well as the delamination peeling, deposit, and backfill of the topsoil.

### 4.4. The Reclamation Model of the Eco-Agriculture Overall Development in Shallow Ponding Collapse

The comprehensive development direction of the shallow ponding collapse is to make full use of the ponding conditions to develop fishing, turning the simple planting agriculture before collapse into an ecological agriculture combing planting and breeding [31].

The major measure is digging deep to fill shallow.

According to the food cycle theory in ecology in this model, many diggings allocate the agriculture, forestry, and herding reasonably, achieving the comprehensive operating for ecological agriculture. In this system, the crops and fodder can be used as feed for stock-raising. The feces of herding can be used as fish bait for aquaculture and also can be used as fertilizer for farmland directly. The pond sludge in fish pond is a good organic fertilizer. Ultimately, the multistage circulation utilization of the material is formed.

### 4.5. The Model of Developing Park Scenery Traveling Reclamation in Collapse

Generally the diggings are developed into mining cities of energy and heavy industry. The mining around is densely populated and residential quarters. Programming reasonably and investing to rest, afforest, and construct recreational facilities for the local collapse to become the place for recreation can not only improve life equality of people in mining but also expand the urban green area [32]. Therefore this model has great significance on improving local environment and biodiversity in mining area.

### 4.6. The Comprehensive Reclamation of Eco-Agriculture in Mining Subsidence

The subsidence of the diggings is the inevitable results of the impaired surface. Multidestruction forms appear on the surface, especially for the adverse effect for agricultural environment and the damage is also obvious. The comprehensive treatment for ecological agriculture in mining is not the simple reclamation but the integrated reclamation process of agriculture, forestry, herding, fishing, and processing industries. It is the combination and assembling according to ecology principle. It makes full use of the symbiotic relationship to allocate plant, animal, and microorganism reasonably to perform vertical farming and aquaculture reclamation. In terms of multilevel energy exploitation and circulating and regenerating mechanism, it is the recycle utilization of waste in agricultural industry, making the agricultural organic wastes to become resources and increasing products. It makes full use of modern science and technology to plan rational to achieve the unification of economy and social and ecological benefit [10]. Therefore, the comprehensive treatment of ecological agriculture in mining collapse is the key link in the comprehensive treatment of the whole mining area of ecological agriculture.

## 5. The Design and Model for Eco-Agricultural Reclamation in Mining Subsidence

### 5.1. The Design of Eco-Agricultural Reclamation in Mining Subsidence

The design of ecological agricultural reclamation in mining subsidence includes graphic design, vertical design, food chain design, time design, and engineering design [27]. In the graphic design and vertical design, it should be noticed that make full use of the specific terrain and land form produced by mining and realize the local hydrogeological and climate conditions to distribute rationally [33]. The spatial arrangement has to meet the requirements for light, heat, water, and air, living up to reasonable, effective, and economic. In the food chain design, we should keep away from the populations which can degrade harmful substance and the populations which do not absorb and enrich harmful substance [9]. In the time design, we should have a good standing of the local resources time rhythm following the ecological agriculture suitability analysis.

The graphic design for ecological agricultural reclamation in mining subsidence is to determine biotic population or ecotype and the proportion distributions occupied by the industrial in a certain reclamation area to achieve the optimal utilization of various resources in horizontal space.

**Figure 5:**
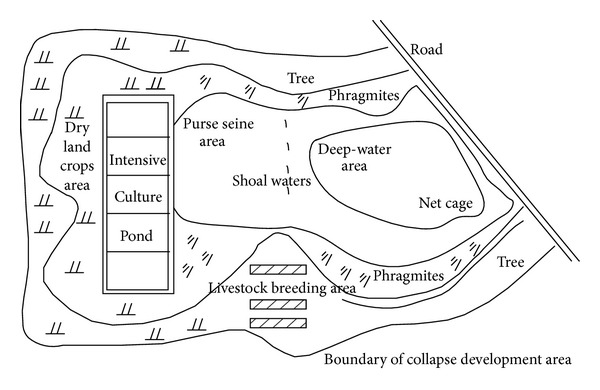
Example of graphic design for ecological agricultural reclamation in mining subsidence.

The example of graphic design for ecological agricultural reclamation in mining subsidence is as shown in Figure 5.

### 5.2. The Model of Eco-Agricultural Reclamation in Mining Subsidence

The model of ecological agricultural reclamation in mining subsidence is a grade separation between time and space. It is a rational combination ecological agricultural reclamation system according to the mutually beneficial relationship between biology and ecology characteristic and biology [34].

Based on the biological form, habitat heterogeneity, and the numbers of abiological factors, the three-dimensional symbiosis of ecological agricultural reclamation can be divided into the following types and modes.

(1) The stereoscopic plantation type:

  
*①* the intercropping, interplanting, and rotation mode, such as grain-cotton, grain-oil, and grain-vegetable;
  
*②* the stereoscopic plantation mode of forest crops. With the different species, the combinations are different;
  
*③* the stereoscopic plantation mode of tree-crop intercropping, such as paulownia-grain intercropping, jujube-grain intercropping, and fruit tree-vegetable intercropping;
  
*④* the stereoscopic plantation mode of tree-medicine intercropping. It refers to the spatial organization of forest, fruiter, and pharmaceutical plant.

(2) The stereoscopic aquaculture type: the stereoscopic aquaculture means the hierarchical configuration of the farmed animals in a certain space or the integrate of products in a certain period of time. The dominate modes are as follows.

  
*①* The stereoscopic captive breeding mode on land: it is a stereoscopic aquaculture of interspace symbiosis so as to make full use of space, save material, and make use of waste. For example, bee barrel (upper layer)—hen house (middle layer)—pigsty (under layer)—earthworm (bottom layer).
  
*②* The stereoscopic aquaculture mode in water: it is a spatial configuration mode in order to make full use of space, nutrition, and dissolved oxygen in water. The usual combinations are chub (upper layer)—grass carp (middle layer)—black carp (under layer); duck (upper layer)—fish (middle layer)—oyster (under layer); fish (upper layer)—trionychidae (under layer).

(3) The stereoscopic cropping and raising as follows.

It refers to planting botany and farming animals in a certain space. It is to form a simple food chain among animals and is characterized by the symbiotic relationship among organisms. The upper plant offers concealment conditions and living environment for the animal and plant in under layer. The free-ranging creature in under layer is able to clear weeds and pests under the crops promoting the growth of upper plant. Meanwhile, the plant or animal waste in under layer provides bait for fish in bottom water, while the activities of fish increase the dissolved oxygen in water, promoting the permeability of plant roots. The dominate modes of this type are as follows: rice—duckweed—fish, rice—duck—fish, forest—livestock—earthworm, reed (as well as other swamp crops)—birds—fish, and so on.

Totally, the suitable tree species and the standard of planting density in Shanxi Province are summarized in Table 3.

## Figures and Tables

**Table 1:** Technical requirements of the land reclamation mode.

Reclamation mode	Application	Technical requirements
Agriculture	Plantation, garden	Land leveling spread topsoil. For the food crops, the topsoil should not be less than 0.5 meter, of which the humus layer should not be less than 0.2-0.3 meter. The filling material should not contain harmful element, if any the isolation layer is needed and the thickness should not be less than 0.4 meter. The hydraulic condition should be good. The demand of topsoil: the soil mass density should not be more than 1.5 g/cm^3^. The proportion of clay and sand is 1 : 3 or 1 : 2. The porosity is no less than 40%–50%. The content of the soluble sodium sulfate and magnesium sulfate is no more than 5%. Sodium oxide is no more than 0.01% and the pH value should be 6–8.

Forestry	Planting trees, orchard	The terrain may have appropriate grade and the topsoil is needed. For planting trees, the topsoil is no less than 0.3 meter and the plant pit needs more than 1 meter. The isolated layer is needed if the filling material contains harmful elements. The thickness of the filling material is no less than 0.4 meter and filling material should be punning.

Fishery	Reservoir, fish-farming	The gradient of the shoreside should not be too steep. The area of the water should not be too large. The quality of the water should meet the water quality standard for fisheries.

Construction	Civilian, industry	The land needs to be punned well and the houses need antideformation measure.

Tourist	Stadium, park, swimming pool	The land needs to be punned well and the houses need antideformation measure.

Headwaters	Irrigation, drinking	The land needs to be punned well. The isolated layer is needed and its thickness is no less than 0.5 meter. In addition, cement to harden the surface is needed if necessary.

**Table 2:** The improvement measures for three typical reclaimed soils.

Reclaimed soil	Defect	Improved measures
Fly ash	Short of organic matter and nitrogenous fertilizer, bad field water performance, powerful heat absorptivity, fast heat, high pH value	Returning straw to field, rotation of green manure, the cooperation of organic and inorganic nitrogenous fertilizer, to transform pouring to sprinkling irrigation, laid straw interval, application of acid fertilizer

Mud	More clay particle, weak permeability, low content of organic matter	Returning straw to field, fly ash improvement, increment in humic and acid fertilizer

Rotten gangue	Low content of available water, strong endothermic character, fast heat, low content of nutrient element	Regular irrigation, keep a full stand of seedlings, laid straw interval, increment in humic and acid fertilizer

**Table 3:** The suitable tree species and the standard of planting density in Shanxi.

Terrain	Chief species	Planting density (plant/mu)
Mountainous and hilly area	Larch, birch	220–330
	Picea meyeri, picea wilsonii, chinese pine, pinus armandi, pinus bungeana, fir, platycladus, oak	330–440
	Sumac	40–70
	Pecan	10–25
	Jujube	16–56

Basin and plain	Poplar, willow, catalpa	80–150
	Paulownia	40–70
	Elm, locust tree	220–330

Remarks	Species of pygmyism and minor, fit for growth in line spacing	
